# Systematic Review and Meta-Analysis of Mercury Exposure among Populations and Environments in Contact with Electronic Waste

**DOI:** 10.3390/ijerph191911843

**Published:** 2022-09-20

**Authors:** Gwen Aubrac, Ashley Bastiansz, Niladri Basu

**Affiliations:** 1 Bieler School of Environment, McGill University, Montreal, QC H3A 2A7, Canada; 2 Faculty of Agricultural and Environmental Sciences, McGill University, Montreal, QC H9X 3V9, Canada

**Keywords:** mercury, electronic waste, metals, review, occupational health

## Abstract

Electronic waste (e-waste) recycling releases mercury (Hg) into the environment, though to our knowledge Hg levels at such sites have yet to be examined on a worldwide basis. A systematic review of scientific studies was conducted to extract, analyze, and synthesize data on Hg levels in e-waste products, environments near recycling sites, and in people. Data were extracted from 78 studies from 20 countries, and these included Hg levels in 1103 electrical and electronic products, 2072 environmental samples (soil, air, plant, food, water, dust), and 2330 human biomarkers (blood, hair, urine). The average Hg level in products was 0.65 μg/g, with the highest levels found in lamps (578 μg/g). Average soil and sediment Hg levels (1.86 μg/g) at e-waste sites were at least eight times higher than at control sites. Average urinary Hg levels (0.93 μg/g creatinine) were approximately two-fold higher among e-waste workers versus control groups. Collectively, these findings demonstrate that e-waste recycling may lead to Hg contamination in environments and human populations in close proximity to processing sites. These findings contribute to a growing knowledge base of mercury exposure through diverse source–exposure pathways, and the work has potential policy implications in the context of the Minamata Convention.

## 1. Introduction

Mercury is a global pollutant that poses a risk to human and environmental health. The chemical form in which mercury and its compounds occur influences its environmental fate and human exposure pathways [1,2,3]. Understanding these processes provides important information as exposure to mercury has the potential to adversely impact the nervous, cardiovascular, and immune systems [2,3].

Mercury may be released in certain industrial settings such as manufacturing processes involving mercury (e.g., polyurethane, chlor-alkali, and acetaldehyde production), artisanal and small-scale gold mining (ASGM), coal-fired power plants and industrial boilers, smelting and roasting processes in the production of non-ferrous metals, waste incineration, and cement production [4]. Another industrial sector that releases mercury into the environment is the recycling of electronic waste (e-waste). E-waste results from the disposal of electrical and electronic equipment (EEE), a term used to describe products with circuits or electrical components and a power or battery supply [5]. E-waste can refer to a range of different devices, including large household appliances (e.g., refrigerators, air conditioners, ovens, dryers, toasters) and consumer electronics (e.g., televisions, stereos, cell phones, computers) [6]. Many types of EEE contain elemental or gaseous mercury, including fluorescent lights, switches, batteries, phones, and computers [5,7]. In fact, it has been estimated that approximately 50 tons of mercury are found in globally undocumented e-waste annually [5]. When these products are dismantled or burned for recycling, mercury can then be released into the environment and have hazardous implications towards human and environmental health [5,8].

The Minamata Convention on Mercury came into force on 16th August of 2017 with the objective of fostering global cooperation in reducing and eliminating the use of mercury to protect human and environmental health from anthropogenic emissions and releases of mercury and mercury compounds [9]. The Convention presents various measures to meet this objective, including regulations on mercury supply sources and trade, mercury-added products, manufacturing processes involving the use of mercury, artisanal and small-scale gold mining (ASGM), environmental emissions and releases, and mercury-containing wastes [9]. Article 16 of the Convention urges parties to develop educational and preventive programs aimed at reducing occupational exposure to mercury [9]. In the context of the rapid growth of e-waste worldwide, and rising concerns about mercury exposure, there is a need to address existing knowledge gaps and provide accurate and up-to-date information about mercury exposure through e-waste activities. Such information can guide future research and policymaking by highlighting mercury sources in e-waste, understanding the potential for environmental contamination, and gauging human health risks to both e-waste workers and nearby inhabitants to recycling sites.

The objective of this systematic review was to compile and synthesize existing information on mercury levels at e-waste sites as well as to characterize mercury exposures among e-waste workers and populations living in proximity to e-waste processing sites. This objective was realized through a systematic search of the peer-reviewed scientific literature. Data from studies, based on pre-defined criteria, were extracted and analyzed both qualitatively and quantitatively to identify consistent areas of knowledge as well as data gaps. A meta-analysis was performed to quantify mercury in the three following groups of interest: (1) “Mercury in e-waste products”; (2) “Mercury in environments contaminated by e-waste”; and (3) “Mercury in human biomarkers of populations exposed to e-waste.”

## 2. Materials and Methods

### 2.1. Literature Search

**Figure 1:**
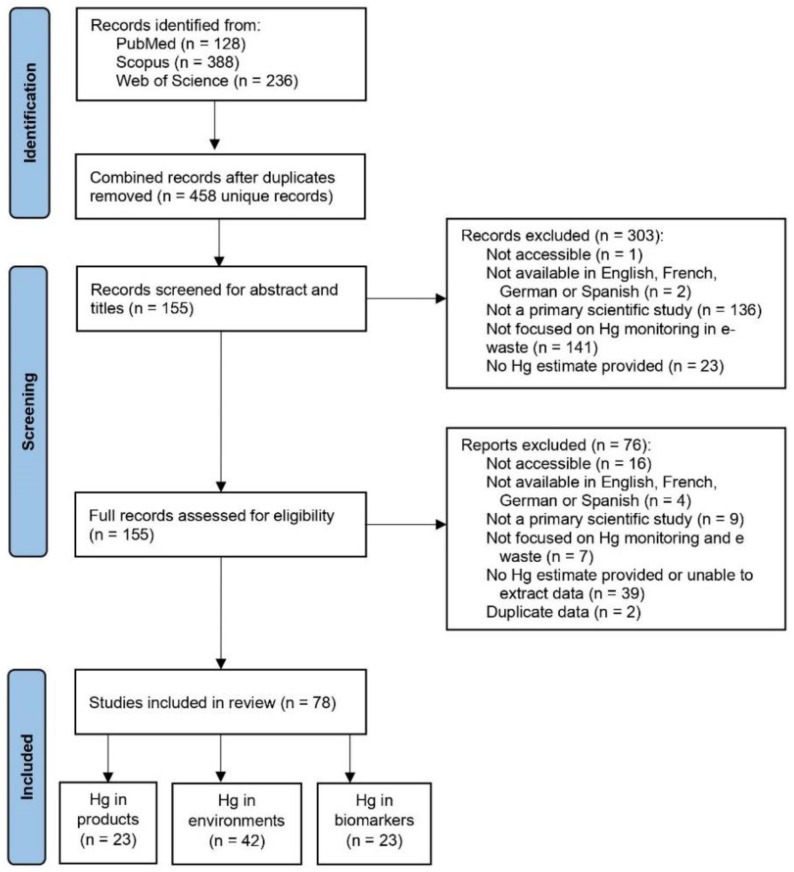
PRISMA chart indicating the number of articles that were identified, screened, and included in this literature review for the three main study groups.

A protocol for the initial search strategy was developed in November 2021. We reviewed key resources [10,11] including a systematic review concerning methylmercury exposure from seafood consumption [12], human biomarkers of mercury exposure [13], and metals pollution at e-waste sites [13]. The literature search (of publications as of 10th January 2022) and screening process are summarized through a PRISMA chart (Figure 1). 

Reports on mercury content in e-waste products and processing sites along with mercury exposure among populations involved with e-waste recycling activities were identified through a systematic search of the peer-reviewed scientific literature. An electronic search was carried out in three databases (PubMed, Scopus, and Web of Science) on 10th January 2022. The search strategy included two Boolean phrases that were combined with AND: #1—(mercury OR methylmercury OR Hg OR MeHg); and #2—(Ewaste OR “electronic waste” OR e-waste OR WEEE OR “waste electrical and electronic equipment”). This search yielded a total of 752 results, of which 128 were from PubMed, 388 were from Scopus, and 236 were from Web of Science. All articles were downloaded to the reference manager Zotero, from which 294 duplicates were identified and removed, resulting in a total of 458 unique articles (Figure 1).

### 2.2. Screening Process

Original articles were reviewed through a two-step screening process. First, titles and abstracts were screened for inclusion based on the following pre-defined criteria: the article was available in English, French, German, or Spanish; the full article was accessible; the article was a primary scientific study; the article was primarily focused on mercury in e-waste, contaminated sites, or populations in contact with e-waste; and the study provided an estimate of the central tendency value or upper boundary value for mercury content, or a measure of variation from which they could be estimated.

Second, 155 articles from the 458 articles identified were included and read in full to further assess their eligibility based on the same aforementioned criteria. A total of 78 articles were included in the final review. Data from included studies were grouped into the following areas of interest (with some papers falling into multiple groups, and the number of included papers noted in brackets): (1) “Mercury in e-waste products” (n = 23); (2) “Mercury in environments contaminated by e-waste” (n = 42); and (3) “Mercury in biomarkers of populations exposed to e-waste” (n = 23). Studies that reported on mercury measurements in food or indoor environments were grouped under the category of “Mercury in environments contaminated by e-waste”.

### 2.3. Data Extraction

From each included paper, data were extracted on the study design (sample type, sample media, sample size, location, WHO region, year of sample collection, year of publication), methods and media for mercury measurement (unit of measurement, instrument used for mercury measurement, detection limit, analytical accuracy, and analytical precision), and mercury measurement values (central tendency value and high-end value). For studies relevant to “Mercury in environments contaminated by e-waste” and “Mercury in human biomarkers of populations exposed to e-waste”, information on control samples (sample size, description) was also extracted. The order of preference for central tendency values to be extracted from a paper was as follows: geometric mean > median > mean. The order of preference for the upper boundary value extracted was: 95th percentile > 90th percentile > 75th percentile > maximum value > two times the standard deviation. All data were compiled into Microsoft Excel and visualized using R [14].

### 2.4. Study Quality

Study quality of the included articles was assessed using the Office of Health and Translation Risk of Bias tool [11] published by the National Toxicology Program in 2019. For the purpose of this review, the following items were considered: (1) quality of measurement instrument reported; (2) accuracy through the use of appropriate reference material; (3) precision through the use of replicate measures; (4) reported limit of detection; (5) selection method; (6) sample size; (7) use of an appropriate control group; (8) demographics reported; (9) mercury exposure characteristics reported; (10) key descriptive measures reported. Items 1–6 were considered for “Mercury in e-waste products”; items 1–7 were considered for “Mercury in environments contaminated by e-waste”; and items 1–10 were considered for “Mercury in human biomarkers of populations exposed to e-waste”. Generally, items were scored “0” when no information was provided, “1” if information provided on the item was unclear or if the item did not meet an adequate a priori threshold, and “2” if the item was reported clearly and met an adequate a priori threshold (Table S1).

For “Mercury in e-waste products”, study quality ratings ranged between 0–12 and were characterized as low quality (0–4), moderate quality (5–8), and high quality (9–12). For “Mercury in e-waste contaminated environments”, study quality ratings ranged between 0–14 and were characterized as low quality (0–4), moderate quality (5–9), and high quality (10–14). For “Mercury in human biomarkers of populations exposed to e-waste”, study ratings ranged between 0–20 and were characterized as low quality (0–6), moderate quality (7–14), and high quality (15–20).

### 2.5. Data Analysis

To optimize cross-study comparisons, our analysis focused on data on “total mercury” concentrations. Studies reporting on organic and inorganic mercury concentrations were considered in the discussion. For example, data on methylmercury in samples from four studies were excluded from the main analysis but considered in the discussion [15,16,17,18]. Total mercury measurements from the analyzed samples were harmonized to the following common units: (1) μg/g for e-waste products and environmental samples (soil, sediments, dust, water, food or animals, plants, and other); (2) μg/m^3^ for environmental air samples; (3) μg/L for blood, serum, and urine samples; (4) μg/g of creatinine for creatinine-adjusted urine samples; and (5) μg/g for hair samples.

In some cases, results were harmonized to dry weight concentration (Cdw) from the reported wet weight concentration (Cww) using the following equation: Cdw = Cww/((100 − W)/100), where W is the water content of the sample [19]. The reported result was divided by the average weight of the sample when the mercury mass was given for all combined samples. The average water content of analyzed samples was estimated using various external sources (Table S2). Similarly, the mercury content in μg per g was calculated based on the average mass of the sample when mercury content was reported as a percentage of the sample.

For studies that reported a mercury concentration below the limit of detection (LOD) in their sample, an estimated value was calculated as the LOD divided by the square root of 2 based on guidance detailed elsewhere [20]. If multiple parts of a same sample were analyzed, the sample size was counted as one rather than the number of different parts analyzed. Similarly, when composite samples were taken from the same area, the sample was averaged into a single value and included as a single sample. When no sample size was given, it was assumed that the sample size was one.

Finally, for environmental and human biomarker samples, data were grouped by exposure categories (Table 1). Samples from sites or populations exposed to e-waste were defined as category 1 when the exposure was direct (e.g., active e-waste site or worker) and category 2 when exposure was indirect (e.g., abandoned e-waste site or nearby resident). Samples from control sites or populations were defined as category 3 when the control was near e-waste activity (e.g., office space or office worker of an e-waste facility) and as category 4 when the control was not exposed to any form of e-waste (e.g., distant site or population of another city).

## 3. Results

### 3.1. Study Location and Quality

This review was based on data contained within 78 peer-reviewed scientific papers published between 2005 and 2022 (Table 2 and Table S3). Most of the studies concerning mercury content in e-waste samples hailed from Europe, whereas mercury in environmental samples and human biomarkers were from the Western Pacific region (Figure S4).

### 3.2. Mercury in E-Waste Products

**Figure 2:**
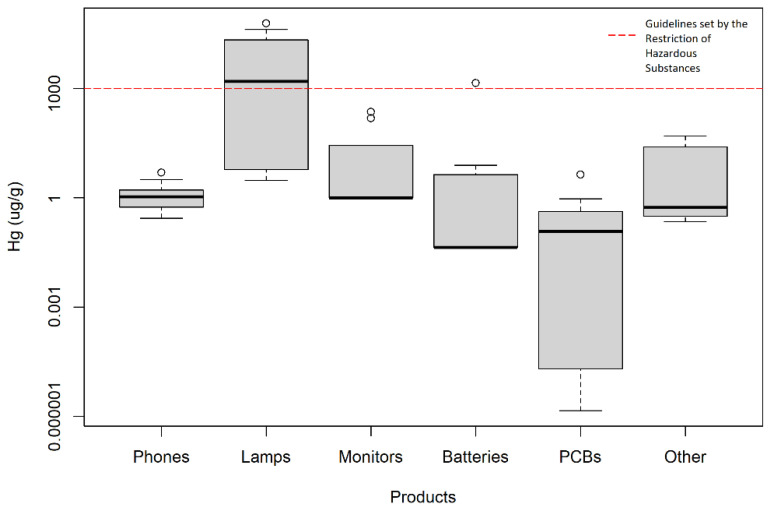
Central Tendency Values of Mercury in E-Waste Products. Results encompass data from 1103 e-waste products: phones (n = 89); lamps (n = 69); monitors, screens, and liquid crystal displays (n = 419); batteries (n = 235); printed circuit boards (n = 259); and other e-waste products (n = 32).

A total of 1103 e-waste products were compiled and analyzed (Figure 2 and Figure S1). The quality of these studies ranged from 1 to 11 with a median score of 6 (Table 2); About three-quarters (74%) of these studies were categorized as moderate quality, with the rest categorized as either low quality (22%) or high quality (4%). Among the 788 samples, 47 (6%) had mercury levels below the limit of detection (LOD) and for which an estimated mercury concentration was calculated from the LOD provided by the authors. A total of 122 samples (15.5%) had no detectable mercury nor reported LOD, thus no estimate could be calculated and the samples were excluded from the analysis.

The most common detection method used to analyze mercury in e-waste product samples was inductively coupled plasma mass spectrometry (ICP-MS) (37.8%) followed by cold vapor atomic absorption spectrometry (CVAAS) (15.8%) and several other methods (Table S4). Mercury detection instruments were not mentioned for 5.5% of the samples. In terms of data analysis, 27.6% of the studies reported mean mercury concentrations, while this information was not specified in 38.6% of the cases. Nearly three-quarters of the studies (72.4%) did not report an upper boundary measurement, and of those that provided some estimate of the highest levels, 21.3% reported on the maximum value and 6.3% used two times the standard deviation (6.3%).

Overall, the geometric mean of the central tendency mercury values of the 1103 sampled e-waste products was 0.65 μg/g (ranging from 0.00000141–60,900 μg/g), while the geometric mean of the upper boundary mercury values of the products was 4.76 μg/g (ranging from 0.34–154,400 μg/g). Looking more specifically at each type of e-waste product, the average mercury levels of the samples could be ranked from highest to lowest as: lamps (578.31 μg/g; n = 69) > phones (0.96 μg/g; n = 89) > batteries (0.41 μg/g; n = 235) > other e-waste products (0.41 μg/g; n = 20) > monitors, screens, and liquid crystal displays (0.16 μg/g; n = 419) > printed circuit boards (0.0082 μg/g; n = 259) (Figure S1). The category of other e-waste products included internet routers, general purpose polystyrene from housing covers of e-waste items, panel glass, plastic housing, wires, solder, ink cartridges, and photovoltaic panels.

### 3.3. Mercury in Environments Contaminated by E-Waste

**Figure 3:**
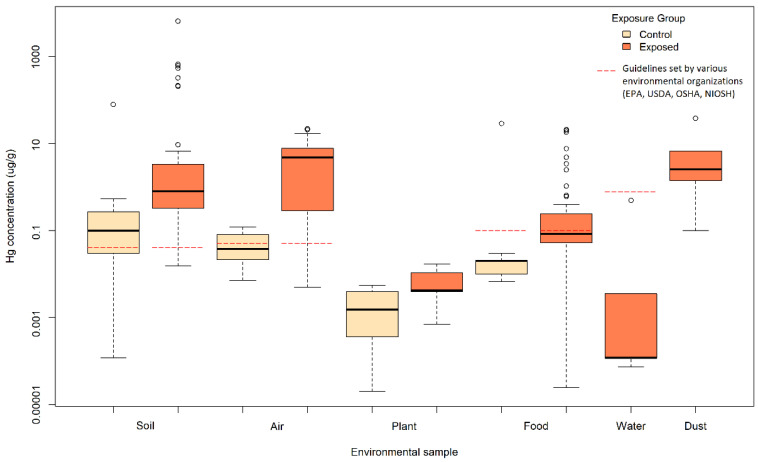
Central Tendency Values of Mercury in Environmental Samples Exposed to E-Waste. The data are represented as boxplots based on data from individual studies (indicated as circles when they are out of the boxplot range). Control sample refer to exposure group 3 or exposure group 4, and exposed samples refer to exposure group 1 or exposure group 2 depending on the data available. Guidelines for each media were extracted from the average European topsoil mercury concentration for soil [21], OSHA and NIOSH for air [22], the USDA for food [23], and the U.S. EPA for water [24].

A total of 2072 samples from environments contaminated by e-waste were compiled and analyzed (Figure 3, Figure S2). The quality of these studies ranged from 3 to 13 with a median score of 9; most of the studies of this data group were categorized as being of moderate quality (52%) or high quality (43%). Among the samples, 36 (1.7%) had mercury concentrations below the LOD, but an estimated concentration was calculated from the provided LOD. A total of 15 samples (0.7%) had no detectable mercury and no reported LOD, and these samples were excluded from the analysis. The most commonly used methods to detect mercury in environmental samples were atomic fluorescence spectrometry (AFS) (28.9%) and cold vapor atomic absorption spectrometry (CVAAS) (28.1%) (Table S4).

The central tendency types of analyzed environmental samples were the mean (34.1%), geometric mean (20.8%), median (5.5%), mercury concentrations from a single sample (7.1%), and the calculated limit of detection over the square root of 2 when no alternative was provided (2.1%). The central tendency type was not specified for 30.3% of the samples. The upper boundary types of analyzed samples were the maximum (22.7%), 2xSD (20.8%) and 95th percentile (0.7%). The upper boundary type was not specified for 55.6% of the samples.

#### 3.3.1. Environments Contaminated by E-Waste: Soil and Sediments

Soil and sediments near e-waste sites were among the most collected type of environmental samples analyzed for their mercury content. Based on results from the meta-analysis, there is a decreasing trend in the average mercury levels in soil and sediment samples as they are less directly exposed to e-waste recycling activities. The same trend was observed for the highest mercury levels detected in samples, with the highest maximum mercury level observed in samples collected on active e-waste sites, followed by abandoned sites or areas with indirect e-waste activity and control sites.

The central tendency value of mercury from 707 soil and sediment samples from active e-waste sites (exposure group 1; 1.86 μg/g) was about 8.5 times higher than levels from abandoned e-waste sites or sites with indirect e-waste activity (exposure group 2; 0.22 μg/g), and at least 20 times higher than levels from controls near e-waste and controls far from e-waste (exposure group 3 and exposure group 4; 0.05 and 0.10 μg/g).

#### 3.3.2. Environments Contaminated by E-Waste: Water

Only four studies examined the mercury content in water samples near e-waste sites and results reveal a higher average mercury content among the control sites rather than the exposed sites. For samples from active e-waste sites (exposure group 1), the central tendency from 45 water samples was obtained with a geometric mean of 0.01 μg/L (ranging from 0.000073–0.49 μg/L), and the upper boundary from 37 water samples was obtained with a geometric mean of 0.0015 μg/L (ranging from 0.001–0.93 μg/L). Then, for control sites not exposed to e-waste (exposure group 4), the central tendencies from eight water samples were obtained with a geometric mean of 0.043 μg/L (ranging from 0.025–0.077 μg/L), which is about four times higher than for the exposed sites (exposure group 1).

#### 3.3.3. Environments Contaminated by E-Waste: Dust

For dust samples, the average mercury level was almost 44 times higher in samples collected on abandoned e-waste sites or sites with indirect e-waste activity (exposure group 2; 2.20 μg/g; n = 27) compared to active e-waste sites (exposure group 1; 2.44 μg/g, n = 66), but nearly five times lower in control sites compared to either type of exposed sites (exposure group 4; 0.05 μg/g, n = 4). On the other hand, maximum mercury levels were highest on sites with direct e-waste activity (exposure group 1; 12.05 μg/g; n = 65) compared to sites with indirect e-waste activity (exposure group 2; 0.66 μg/g; n = 23).

#### 3.3.4. Environments Contaminated by E-Waste: Food and Animals

The average mercury level of food and animal samples was highest on potentially exposed control sites (exposure group 3; 0.17 μg/g; n = 10), followed by sites with indirect e-waste activity, (exposure group 2; 0.15; n = 13), direct e-waste activity (exposure group 1; 0.12 μg/g; n = 198), and unexposed control sites (exposure group 4; 0.04 μg/g; n = 78). Mercury levels among samples from sites actually or potentially exposed to e-waste (exposure groups 1 through 3) were similar and at least three times higher compared to samples obtained from unexposed control sites (exposure group 4).

#### 3.3.5. Environments Contaminated by E-Waste: Air

Air was also among the most analyzed type of environmental sample collected on sites involved with e-waste recycling. The average mercury level in air samples from sites directly involved with e-waste (exposure group 1; 1.29 μg/m3; n = 774) were around 2900 times higher compared to potentially exposed control sites (exposure group 3; 0.0004 μg/m3; n = 6) and 38 times higher compared to unexposed control sites (exposure group 4; 0.03 μg/m3; n = 114).

#### 3.3.6. Environments Contaminated by E-Waste: Plants

Plant samples collected on sites with direct e-waste activity (exposure group 1; 0.0047 μg/g; n = 194) had the highest average mercury content of all sites and these were about 27 times higher compared to unexposed control sites (exposure group 4; 0.00066 μg/g; n = 38) and 7 times higher compared to sites with indirect e-waste activity (exposure group 2; 0.00017 μg/g; n = 14). However, plant samples collected from unexposed control sites (exposure group 3) were 3.8 times higher compared to plants sampled on sites with indirect e-waste activity (exposure group 2).

#### 3.3.7. Environments Contaminated by E-Waste: Summary

Overall, there is a general decreasing trend in the average (Table 3) and maximum (Table S5) mercury levels in environmental samples as the extent of e-waste activity on the site where they were collected decreases. However, there are some exceptions to this trend. More specifically, water samples were highest in control sites compared to exposed sites. Furthermore, dust, food and animal, and plant samples collected on indirectly exposed sites were also generally higher than directly exposed sites. In terms of geometric means, the samples indirectly contaminated by e-waste (exposure group 2) were around 12% the value of those from directly contaminated environments (exposure group 1) for soils and sediments, 90% for dust, and 130% for food and animals. The control samples potentially contaminated by e-waste (exposure group 3) were approximately 3% of the value of those from directly contaminated environments (exposure group 1) for soils and sediments, 146% for food and animals, and 0.03% for air. Finally, the control samples not exposed to e-waste (exposure group 4) were approximately 5% the value of those from directly contaminated environments (exposure group 1) for soils and sediments, 3900% for water, 31% for food and animals, 3% for air, and 14% for plants.

### 3.4. Mercury in Biomarkers of Populations Exposed to E-Waste

**Figure 4:**
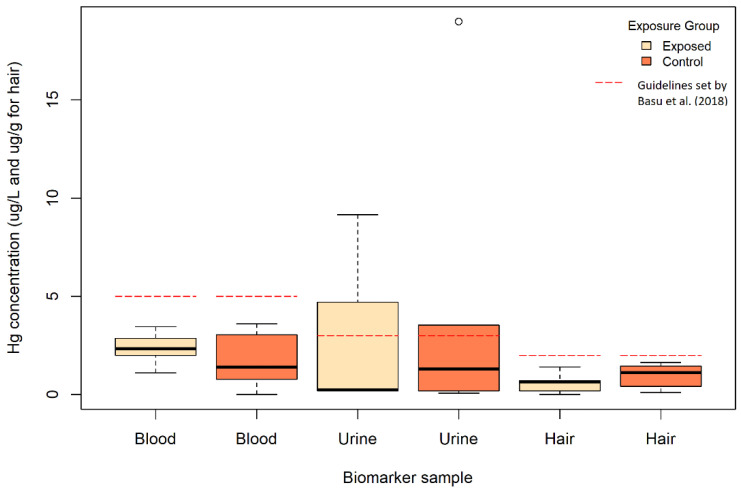
Central Tendency Values of Mercury in Biomarker Samples of Populations Exposed to E-Waste.

A total of 2330 unique individuals exposed to e-waste and controls were sampled for mercury in biomarkers, of which 37 samples (1.6%) had mercury levels below the LOD but an estimated concentration was calculated from the provided LOD (Figure 4, Table 4, Figure S3). The measurement methods used to analyze human biomarker samples were ICP-MS (28.1%), other (28.1%), CVAAS (25%), MA (12.5%), and AFS (1.6%) (Table S4). The measurement instrument used to detect mercury was not mentioned for 4.7% of the samples. The central tendency types of analyzed samples were mean (43.8%), median (24.2%), geometric mean (23.4%), and LOD/sqrt (2) (0.8%). The central tendency type was not specified for 7.8% of the samples. The upper boundary types of analyzed samples were: 2xSD (35.9%), maximum (33.6%), 95th percentile (12.5%), 75th percentile (3.9%), and 90th percentile (3.1%).

Throughout the analysis, exposure group 1 refers to individuals directly involved with e-waste, such as workers in indoor or outdoor e-waste sites. Exposure group 2 refers to individuals indirectly involved with e-waste, such as persons living in proximity to e-waste sites or retired e-waste workers. Exposure group 3 refers to individuals in control groups that may have been exposed to e-waste, such as control workers from an e-waste workshop or a site that are not directly involved with e-waste or controls from a city in which there is e-waste activity. Finally, exposure group 4 refers to individuals in control groups who have never been involved with e-waste and were sampled from a city distant from e-waste activity.

#### 3.4.1. Blood and Serum Samples

The blood mercury level from sampled e-waste workers (exposure group 1; 0.60 μg/L; n = 399) was about two times lower than the blood mercury level of indirectly exposed e-waste workers (exposure group 2; 1.30 μg/L; n = 515) and four times lower than the potentially exposed controls (exposure group 3; 2.60 μg/L; n = 116) and unexposed controls (exposure group 4; 2.22 μg/L; n = 409) (Table 4).

The serum mercury levels of e-waste workers (exposure group 1; 0.70 μg/L; n = 128) was nearly the same as that of workers indirectly exposed to e-waste (exposure group 2; 0.8 μg/L; n = 26) and potentially exposed controls (exposure group 3; 0.8 μg/L; n = 65).

#### 3.4.2. Urine Samples

The urine mercury level from e-waste workers (exposure group 1; 0.10 μg/L; n = 273) was 260 times higher compared to indirectly exposed workers (exposure group 2; 0.0004 μg/L; n = 11), 6 times lower compared to potentially exposed controls (exposure group 3; 0.66 μg/L; n = 10), and 3 times lower compared to unexposed controls (exposure group 4; 0.33 μg/L; n = 60) (Table 4).

In terms of the urine mercury level adjusted for creatinine of exposed e-waste workers (exposure group 1; 0.93 μg/g creatinine; n = 324), the value was 2.2 times higher compared to indirectly exposed e-waste workers (exposure group 2; 0.42 μg/g creatinine; n = 26), 1.7 times higher compared to potentially exposed controls (exposure group 3; 0.55 μg/g creatinine; n = 132), and 2.1 times higher compared to unexposed controls (exposure group 4; 0.43 μg/g creatinine; n = 96).

#### 3.4.3. Hair Samples

The hair mercury level of exposed e-waste workers (exposure group 1; 0.72 μg/g; n = 151) was 1.8 times higher compared to indirectly exposed workers (exposure group 2; 0.39 μg/g; n = 227) and around 4 times lower compared to unexposed workers (exposure group 3; 1.00 μg/g; n = 213) (Table 4).

#### 3.4.4. Biomarkers Summary

In terms of geometric means, the biomarker samples from populations indirectly exposed to e-waste (exposure group 2) were around 217% the value of those from biomarkers of populations directly exposed to e-waste (exposure group 1) for blood and 54% for hair. For populations potentially exposed to e-waste, the mercury values were around 433% the value of those from biomarkers of populations directly exposed to e-waste (exposure group 1) for blood and 59% for urine adjusted for creatinine. Finally, the mercury values of control populations unexposed to e-waste (exposure group 4) were around 368% the value of those from biomarkers of populations directly exposed to e-waste (exposure group 1) for blood, 322% for urine, 46% for urine adjusted for creatinine, and 139% for hair.

#### 3.4.5. Comparison with Guidelines

We compared the biomarker data with mercury levels typically found in background populations with no major sources of exposure from the 2018 UN Global Mercury Assessment: 5 μg/L for blood, 3 μg/L for urine, and 2 μg/g for hair [4].

First, among active e-waste workers (exposure group 1), the percentage of studies for which the central tendency value of mercury in sampled persons exceeded the background values set by the 2018 UN Global Mercury Assessment [4] was 0% for blood, 18% for urine, and 0% for hair. In terms of the maximum levels of mercury observed in these groups of active e-waste workers, the percentage exceeding the background values was 44% for blood, 36% for urine, and 0% for hair.

## 4. Discussion

The implementation of the Minamata Convention on Mercury in 2017 created international momentum to address the issue of mercury pollution and its impacts on human and environmental health [9]. E-waste is among the fastest growing waste streams in the world, and it releases tons of mercury every year [5,25]. Understanding the pathways of exposure to mercury through contact with e-waste, or environmental sites contaminated by e-waste, can inform the development of effective policies and interventions to protect human and environmental health. In this review, 78 studies reporting on quantities of mercury in e-waste products, in associated sites, and relevant populations were compiled and analyzed. Overall, mercury content in most of the analyzed e-waste product samples abided to the guidelines set by the Restriction of Hazardous Substances Directive (RoHS) in 2006 [26].

### 4.1. Mercury in E-Waste Products

Minimal attention was devoted to WEEE until 2002 when the first European legislation related to WEEE, Directive 2002/96/EC, was put in place [6]. In 2006, the Restriction of Hazardous Substances Directive (RoHS) was implemented in Europe to monitor the number of toxic metals in electronic and electrical devices, proposing a maximum permissible limit of mercury in electronic and electrical products as 1000 ppm, which is equivalent to 1000 μg/g [26]. This led to studies examining the concentration of hazardous substances, including mercury, in various EEE items. In terms of central tendency values, all analyzed e-waste products adhered to the guidelines set by the RoHS except for fluorescent lights and batteries. One study examining six different fluorescent lamp samples measured mercury above the guideline for all samples, reaching up to 60,900 μg/g [27]. For batteries, one study found a mean mercury content of 1390 μg/g in sampled batteries [28]. In terms of upper boundary values, fluorescent lamps were found to exceed this threshold, reaching a maximum value of 154,400 μg/g [27].

The type of e-waste containing the highest levels of mercury were fluorescent lights. Nearly 80% of the total mercury in fluorescent lights is contained in the phosphorous powder of the device, most of which is vaporized and lost to ventilation during the dismantling process [29,30]. In new light tubes, most of this mercury is in the Hg^0^ form (elemental mercury), as opposed to the Hg^+^ (mercury (I) ion) or Hg^2+^ (mercury (II) ion) form in spent tubes [27]. Mercury vapors are often emitted as soon as lamps are opened, broken, or crushed [31]. While sampled LCD screens were all found to abide to RoHS guidelines, one study mentioned the complications and risks associated with recycling appliances equipped with LCD screens as they have mercury-containing backlights that are difficult to capture during treatment due to the volatility of the mercury [32]. Mercury vapours pose a health risk for recycling plant workers since up to 80% of inhaled mercury vapour is absorbed into the blood, where it can cross the blood–brain barrier and may cause damage to the central nervous system [3,28].

### 4.2. Mercury in Environments Contaminated with E-Waste

Studies commonly reported elevated mercury concentrations in environmental samples near e-waste activity when compared to controls, reference guidelines, or background values. In some cases, the extent of mercury contamination within e-waste sites was significantly higher compared to controls or background values. For example, in the e-waste processing town of Longtang, China, soil and sediment samples were 10 times above the permissible mercury concentration according to environmental quality standards [33]. In Wenling, China, mercury was reported by the researchers to be the most serious metal pollutant in the area, with concentrations exceeding the Grade II value of soil quality standards from the State Environment Protection Administration of China of 0.3 μg/g by 1 to 31 times, except on certain sampling sites [34]. In other cases, mercury levels on e-waste sites were within recommended guidelines presented by the study but higher compared to control sites. For example, in Douala, Cameroon, mercury concentrations in soil samples were 10-fold higher on the e-waste site compared to the control site but were still within the guideline values set by the Finnish legislation, which provide an approximation of mean values in Europe and are used internationally [35].

Environmental mercury concentrations varied depending on several factors. First, mercury concentrations varied according to the type of e-waste activity occurring on site. In Qingyuan, China, soil mercury concentrations were ten-fold higher in the acid-leaching site compared to the abandoned e-waste processing site [36]. In the industrial city of Dongguan, China, soil mercury concentrations were approximately two-fold higher in the dump site compared to the acid-leaching site [37]. In a Swedish e-waste recycling facility, mercury in personal air samplers from workers in the dismantling process and indoor workers were three orders of magnitude higher compared to outdoor workers, suggesting the importance of adequate ventilation in indoor recycling workshops [38]. In the distribution center of a material recycling site in Wen’an, China, the plastic recycling area was particularly contaminated with mercury and exceeded background values by approximately 20 times, though there was a significant variation in concentrations across all areas sampled [39]. Finally, in an indoor recycling facility in Norway, gaseous mercury was attributed to broken devices such as fluorescent bulbs and tubes [40].

Mercury contamination also depended on the type of e-waste processing facility in which the waste was handled and the contamination mechanism through which mercury was released into surrounding environments. For example, surface and air dust in formal e-waste processing sites in Bangalore, India, were not polluted with mercury, but informal sites such as slums were moderately to severely contaminated [41]. In agricultural soil in Taizhou, China, mercury contamination was thought to result from aerial deposition given the low correlation between metal content in rice, hull, and soil samples [42]. In a study of e-waste recycling workshops across Germany, mercury was detected in 70% of the samples and concentrations varied significantly across different recycling facilities, which may have been explained by the use of different occupational safety measures [43]. In a study on mercury in soils from Longtang, China, mercury was not typically associated with other metals, suggesting that contamination came from point sources [44].

Several studies reveal that environmental mercury contamination can make its way into locally grown products, notably rice [45]. Near a compact fluorescent light manufacturing site, the mean total mercury and methylmercury in locally cultivated rice was two times higher than in commercial rice, suggesting that the manufacturing activities may have resulted in mercury accumulation in local rice samples [15]. Similarly, mercury was found to accumulate in plant roots compared to shoots [46]. Such findings may be useful for future risk assessments of mercury exposure through local food consumption.

To put these findings into perspective, the average topsoil mercury concentration in Europe is 0.04 μg/g, with a range from 0 to 159 μg/g [21]. The geometric mean soil mercury concentration in e-waste sites included in this review exceeded this concentration by two orders of magnitude for directly contaminated sites (0.0152 to 6402 μg/g) and one order of magnitude for indirectly contaminated sites (0.00026 to 9.9 μg/g), and were within the same order of magnitude for potentially exposed control sites (0.026 to 0.16 μg/g) and unexposed controls (0.00012 to 78.1 μg/g).

The U.S. EPA’s National Recommended Water Quality Criteria suggests the maximum permissible mercury concentrations in water as 1.4 μg/L for acute exposure and 0.77 μg/L for chronic exposure [24]. All the water samples in this review were below these recommended guidelines (0.000073 to 0.49 μg/L). According to the USDA Maximum Levels of Contaminants in Foods [23], the maximum permissible mercury limit in various types of food items ranges from 0.01 μg/g (vegetables, milk products) to 0.1 μg/g (salt, fungi). Mercury concentrations in food samples exceeded this range in contaminated (0.000025 to 20.8 μg/g) and potentially contaminated sites (0.059 to 0.52 μg/g) while remaining within the same order of magnitude and were within this range for foods sampled in potentially exposed control sites (0.10 to 0.28 μg/g) and unexposed control sites (0.0067 to 28.4 μg/g). Finally, the Occupational Safety and Health Administration (OSHA) set recommended exposure limits at 0.1 mg/m^3^ over an 8-h work shift and the National Institute for Occupational Safety and Health (NIOSH) recommends exposure to inorganic mercury vapor to be within 0.05 mg/m^3^ over a 10-h shift [22]. The air mercury concentration of studies included in this review exceeded these guidelines by two orders of magnitude in locations exposed to e-waste activity (0.005 to 21.7 μg/m^3^) but were within these guidelines in potentially exposed control locations (0.00039 to 0.0005 μg/m^3^) and unexposed control locations (0.0072 to 0.12 μg/m^3^).

### 4.3. Mercury in Biomarkers of Populations Exposed to E-Waste

Across the included studies, accepted biomarkers for assessing mercury exposure in individuals were employed. While some studies found populations exposed to e-waste to have mercury biomarker concentrations within guideline ranges, many of the studies reported on mercury exposures in exceedance of guidelines or controls as summarized here. Pre-school children living in proximity to e-waste activity in Guiyu, China, all had blood mercury levels that exceeded the U.S. EPA guideline of 5.8 μg/L [47]. E-waste recyclers in a Swedish facility had urinary mercury levels higher than measured in a control group of office workers [38]. In the e-waste recycling site of BanKok and the town of BanKlang, Thailand, urinary mercury was higher in participants involved with e-waste recycling compared to those not handling e-waste [48]. Hair mercury concentrations were higher among compact fluorescent light workers in Gaohong, China, compared with control residents [45]. Blood mercury concentrations were higher among residents near the Luqiao e-waste recycling area, China, compared to controls in Huangyan, China, despite the e-waste facilities having been shut down for two years [47]. Finally, blood mercury levels among children from the e-waste recycling area of Guiyu, were found to be higher among those exposed to e-waste, and the levels were associated with alterations in neutrophils, IL-1beta, IL-6, and IL-1RA [49].

In many cases, dietary mercury intake was suspected to be associated with the mercury biomarkers, making it important to account for dietary factors when conducting biomonitoring studies. For example, dietary mercury intake was suspected to be linked to a non-significant increase in mercury exposure among controls in Agbogbloshie, Ghana [50]. In Kumasi, Ghana, higher blood mercury among participants was associated with blood selenium and arsenic, thus suggesting that this increase may have been linked to the consumption of products of marine origin [51]. In Taizhou, China, dietary intake of rice and fish was reported as a major source of mercury exposure among residents sampled in the exposed and control e-waste recycling sites [18]. In a Swedish recycling facility, blood mercury concentrations (unlike the urine ones) were similar between recycling workers and office workers, most likely due to an influence of dietary mercury [38]. Finally, in Hung Yean, Vietnam, members of the control population had higher methylmercury levels compared to female e-waste recyclers, which was indicative of increased fish consumption [17].

In reviewing the studies, several factors were associated with increased mercury levels among populations exposed to e-waste. First, workers involved in different segments of the e-waste recycling process may be exposed to varying levels of mercury. For example, in electronic scrap recycling facilities in the U.S., no employees had detectable mercury in their urine and only one employee had detectable mercury on their skin, but mercury was found on employees’ street clothes in the light bulb recycling areas [52]. Second, mercury exposure typically increased with time spent near e-waste activities. In Kumasi, Ghana, workers who had spent more years and longer hours working at the e-waste facility had higher urinary mercury levels [51]. Additionally, workers using masks and gloves had lower urinary mercury levels, emphasizing the importance of using personal protective equipment [51]. Among workers in the e-waste recycling area of Taizhou, China, hair mercury levels were correlated with the daily numbers of hours spent in the industrial area [18]. Similarly, in Agbogbloshie, Ghana, increased blood mercury concentrations in e-waste workers were correlated to the number of years working at the e-waste site [53]. Third, mercury biomarker levels varied based on the exposure route. Earlier we reviewed the importance of dietary exposures and methylmercury exposures (as measured in blood and urine) and contrasted this with exposures to elemental and inorganic mercury that would be more typically found within e-waste recycling (and be reflected in urinary mercury). In addition, a correlation was found between airborne mercury and urinary mercury among workers of an e-waste recycling facility in the Nakhon Si Thammarat Province, Thailand, although none of the workers displayed urinary mercury levels above guideline levels [54]. In Bangalore, India, mercury in the hair of male workers was non-significantly elevated compared to female workers, possibly because male workers may have been in direct contact with or inhaled mercury during the metal extraction process, which was not carried out by female workers [41]. Lastly, dietary influences other than seafood consumption may also impact mercury in biomarkers. Hair methylmercury concentrations were higher among residents consuming rice cultivated near a compact fluorescent light manufacturing plant compared to those consuming commercial rice in Gaohong, China [45].

The 2018 UN Global Mercury Assessment reported blood, hair, and urinary mercury concentrations among the certain groups exposed to point sources of mercury [4]. Based on findings from this review, median blood mercury concentrations among e-waste workers were 1.4 μg/L for central tendency mercury measurements and 7.4 μg/L for upper boundary mercury measurements. As such, central tendency blood mercury levels among e-waste workers were lower than blood mercury levels of artisanal and small-scale gold mine (ASGM) workers (10 μg/L), people associated with contaminated sites (4 μg/L), and dental workers (3.5 μg/L) based on the findings from the Global Mercury Assessment [4]. Then, median hair mercury levels among e-waste workers compiled in this review were 1.13 μg/g for central tendency mercury measurements and 2.1 μg/g for upper boundary mercury measurements. Considering central tendency hair mercury levels, this means that e-waste workers displayed levels lower than those of ASGM workers (2.5 μg/g) and higher than contaminated site workers (1 μg/g) and dental workers (0.6 μg/g) [1]. Finally, urine mercury levels among e-waste workers compiled in this review displayed a median central tendency value of 0.38 μg/L and a median upper boundary value of 1.7 μg/L. Considering central tendency values, the median urinary mercury levels were lower than urinary mercury levels of ASGM workers (5.9 μg/L), contaminated site workers (3 μg/L), and dental workers (1.2 μg/L) [4]. Overall, the mercury concentrations reported here for e-waste workers were generally lower than those associated with ASGM, contaminated sites, and dentistry.

### 4.4. Limitations

Despite the validity of the methods employed to compile and analyze the data from included studies, limitations related to the cited studies may compromise the quality of the analyses. In terms of our assessment of study quality (Table S1), the most common shortcoming of studies was the sampling method, as all samples were conveniently sampled to examine mercury levels in e-waste products or exposed environments and populations. Another common shortcoming was the failure to report limits of detection of the equipment employed to analyze mercury in samples. As a result, when no mercury was detected in samples, no estimated mercury concentration for the sample could be calculated based on the detection limit. Some studies were imprecise in their reporting of sample size, and researchers were ambiguous about the exact sample size. Most studies examining mercury in e-waste products had low sample sizes, often less than 20 samples. Other studies did not report on their findings thoroughly. For instance, two studies included control groups but did not report the findings for them [55,56]. Similarly, another study reported mercury concentrations only as inequalities, without specifying what the concentrations represented [57].

The geographic extent of studies monitoring mercury, whether in products, environments or biomarkers was limited. For the most part, studies were conducted in China, the United States, and a few select European, Southeast Asian, and African countries. This leaves significant data gaps on the potential exposure to mercury through e-waste across the rest of the globe, particularly in low- and middle-income countries.

This review employed a systematic approach to identify all published studies reporting on mercury exposure through e-waste. Despite the validity of the methods employed to identify and assess the quality of included studies, the findings from the present review are based on the quality of measurements and study designs of included studies. Furthermore, the search was largely restricted to English language databases even though the topic is relatively well-studied in China, for example, which has databases such as CNKI and Wanfang.

### 4.5. The Minamata Convention

These findings suggest several implications in the context of the Minamata Convention. First, most e-waste products, largely sampled in Europe, adhered to guidelines set by the RoHS. However, certain types of e-waste products pose risks of harmful mercury exposure, namely fluorescent lights, batteries, and LCDs. In addition, the distribution of electronic items (including e-waste) is global and often difficult to track, with disproportional differences between the global north and south. Second, most e-waste recycling sites displayed elevated mercury concentrations, sometimes orders of magnitude above national guidelines or background levels. The distribution of mercury was variable within contaminated sites and depended on the type of activity carried out and point sources of mercury. For example, in indoor recycling facilities, the type of recycling activity performed, ventilation infrastructure, and the use of personal protective equipment could lead to different levels of mercury exposure among workers. This highlights the importance of building adequate recycling facilities and enforcing occupational safety measures for workers. Finally, environmental mercury may find its way into locally grown products (i.e., rice), which can have downstream implications on dietary mercury exposure among populations consuming such food items, including both e-waste workers and local residents. In terms of vulnerable populations, the findings from this review suggest that e-waste recyclers and populations, including children, living near e-waste activity could be at an increased risk of mercury exposure.

## 5. Conclusions

This review of 78 publications concludes that e-waste recycling activity may lead to mercury contamination in environments and populations in close proximity to e-waste processing sites. The e-waste products posing the most serious threat for contamination are fluorescent lights, batteries, and LCD backlights. In general, mercury concentrations in e-waste sites exceeded guidelines for soil, local food, and air. Based on the analyses from this review, populations exposed to e-waste generally displayed lower concentrations of mercury in blood, hair, and urine compared to ASGM workers, contaminated site workers, and dental workers with the exception of hair mercury levels, which exceeded those of contaminated workers and dental workers [4]. In terms of maximum exposure, mercury levels in biomarkers often exceeded background values, especially for blood and urine. The findings of this review are limited by the varying quality and narrow geographic extent of studies. This review contributes to a growing knowledge base of mercury exposure through e-waste and presents relevant implications in the context of the Minamata Convention.

## Figures and Tables

**Table 1:** Mercury Exposure Group Categories.

	Environmental Samples		Biomarker Samples	
Category	Description	Example	Description	Example
1	Active e-waste recycling area	Burning site, dismantling site, e-waste workshop, acid-leaching site, shredding site, recycling site, sorting site, food grown in e-waste area	Active e-waste worker	
2	Abandoned e-waste area or near active e-waste area	Commercial site, storage space, desoldering space, loading area, municipal solid waste that may contain e-waste	Person living near e-waste activity or retired e-waste worker	Traders, persons from cities with high e-waste recycling activity
3	Control area near e-waste recycling site	Area surrounding e-waste, office space of e-waste workshop	Person (control) near e-waste activity	Office workers of e-waste workshop, control from a city with high e-waste activity
4	Control area far from e-waste recycling site	Different city, specifically uncontaminated sample	Person (control) far from e-waste activity and never involved with e-waste	

**Table 2:** Studies Included in Data Groups of Interest.

Data Group	Mercury in E-Waste Products	Mercury in Environments Contaminated by E-Waste	Mercury in Biomarkers of Populations Exposed to E-Waste
# Publications	n = 23	n = 42	n = 23
Year Range	2005–2022	2008–2021	2009–2021
Countries	Austria, Belgium, Brazil, China, France, Germany, Greece, India, Iran, South Korea, Sweden, Switzerland, United States	Cameroon, China, France, Germany, Ghana, Greece, India, Indonesia, Nigeria, Norway, Sweden, Thailand	China, Germany, Ghana, India, Indonesia, Sweden, Thailand, United States, Vietnam
WHO Regions	Europe (n = 10), WesternPacific (n = 5), Americas (n = 4), Southeast Asia (n = 1), Eastern Mediterranean (n = 1), not specified (n = 2)	Western Pacific (n = 25), Africa (n = 7), Europe (n = 6), Southeast Asia (n = 4)	Western Pacific (n = 10), Africa (n = 5), Southeast Asia (n = 4), Americas (n = 2), Europe (n = 2)
^1^ Median Study Quality Score	6 [1–11]	9 [3–13]	14 [9–19]

^1^ Possible study quality scores ranged from 0 to 12 for “Mercury in E-Waste Products”, 0 to 14 for “Mercury in Environments Contaminated by E-Waste”, and 0 to 20 for “Mercury in Biomarkers of Populations Exposed to E-Waste”. The numbers in the square brackets indicate the range of study quality scores.

**Table 3:** Central Tendency Mercury Values of Environmental Samples Collected near E-Waste Sites.

Exposure Group		Central Tendency Value								
1	sample media category	unit	total samples	geometric mean	median	mean	IQR	minimum	maximum	SD
	soil and sediment	mg/kg	707	1.86	0.80	204.04	2.87	0.02	6402.00	969.52
	water	mg/kg	45	0.00	0.00	0.10	0.00	0.00	0.49	0.22
	dust	mg/kg	66	2.44	2.54	8.48	4.33	0.10	37.60	14.44
	food or animals	mg/kg	198	0.12	0.08	1.55	0.18	0.00	20.80	4.59
	air	μg/m^3^	774	1.29	4.70	5.87	7.23	0.01	21.70	6.49
	plants	mg/kg	194	0.00	0.00	0.01	0.01	0.00	0.02	0.01
	other	mg/kg	6	7	N.A.	7	N.A.	N.A.	N.A.	N.A.
2	soil and sediment	mg/kg	385	0.22	0.30	0.98	0.54	0.00	9.91	1.94
	dust	mg/kg	27	2.20	2.26	26.75	6.26	0.15	148.00	59.47
	food or animals	mg/kg	13	0.15	0.11	0.23	0.23	0.06	0.52	0.25
	air	μg/m^3^	1	0.035	N.A.	0.04	N.A.	N.A.	N.A.	N.A.
	plants	mg/kg	14	0.00017	N.A.	0.00	N.A.	N.A.	N.A.	N.A.
3	soil and sediment	mg/kg	26	0.05	0.04	0.07	0.07	0.03	0.16	0.07
	food or animals	mg/kg	10	0.17	0.19	0.19	0.09	0.10	0.28	0.13
	air	μg/m^3^	6	0.00	0.00	0.00	0.00	0.00	0.00	0.00
4	soil and sediment	mg/kg	58	0.10	0.10	5.71	0.21	0.00	78.10	20.84
	water	mg/kg	8	0.04	0.05	0.05	0.03	0.03	0.08	0.04
	dust	mg/kg	4	0.05	N.A.	0.05	N.A.	N.A.	N.A.	N.A.
	food or animals	mg/kg	78	0.04	0.02	3.17	0.01	0.01	28.40	9.46
	air	μg/m^3^	114	0.03	0.04	0.05	0.03	0.01	0.12	0.05
	plants	mg/kg	38	0.00	0.00	0.00	0.00	0.00	0.01	0.00

**Table 4:** Central Tendency Mercury Values of Biomarker Samples from Populations Involved with E-Waste.

Exposure Group	Central Tendency									
1	sample media category	unit	total samples	geometric mean	median	mean	IQR	minimum	maximum	SD
	blood	μg/L	399	0.60	1.40	1.81	2.26	0.00	3.60	1.47
	serum	μg/L	128	0.70	0.70	0.70	0.00	0.70	0.70	0.00
	urine	μg/L	273	0.10	0.38	0.50	0.30	0.00	1.40	0.54
	urine adjusted for creatinine	μg/g creatinine	324	0.93	1.31	4.23	3.01	0.07	18.98	7.36
	hair	μg/g	151	0.72	1.13	0.97	0.98	0.10	1.64	0.58
2	blood	μg/L	515	1.30	3.62	3.71	2.07	0.00	11.13	3.32
	serum	μg/L	26	0.8	N.A.	0.80	N.A.	N.A.	N.A.	N.A.
	urine	μg/L	11	0.0004	N.A.	0.00	N.A.	N.A.	N.A.	N.A.
	urine adjusted for creatinine	μg/g creatinine	26	0.42	N.A.	0.42	N.A.	N.A.	N.A.	N.A.
	hair	μg/g	227	0.39	0.88	0.85	0.15	0.01	1.52	0.54
3	blood	μg/L	116	2.60	3.42	2.97	1.55	1.20	4.30	1.60
	serum	μg/L	65	0.8	N.A.	0.8	N.A.	N.A.	N.A.	N.A.
	urine	μg/L	10	0.66	N.A.	0.66	N.A.	N.A.	N.A.	N.A.
	urine adjusted for creatinine	μg/g creatinine	132	0.55	0.24	2.45	2.28	0.18	9.15	4.47
	blood	μg/L	409	2.22	2.34	2.35	0.69	1.10	3.46	0.80
4	urine	μg/L	60	0.33	0.34	0.34	0.01	0.33	0.34	0.01
	urine adjusted for creatinine	μg/g creatinine	96	0.43	0.61	0.61	0.43	0.18	1.04	0.61
	hair	μg/g	213	1.00	1.06	1.06	0.34	0.72	1.40	0.48

## Data Availability

All data are available in the Supporting Information.

## Supplementary Materials

The following supporting information can be downloaded at: https://www.mdpi.com/article/10.3390/ijerph191911843/s1, Figure S1. Upper Boundary Values of Mercury in E-Waste Products; Figure S2. Upper Boundary Values of Mercury in Environmental Samples Exposed to E-Waste; Figure S3. Upper Boundary Values of Mercury in Human Biomarkers of Populations Exposed to E-Waste; Figure S4. Geographic Location of Studies. (a) Location of Studies on Mercury in E-Waste Products; Table S1. Risk of Bias Assessment Methodology; Table S2. Methodology for Conversion from Wet Weight to Dry Weight; Table S3. List of included studies; Table S4. Mercury Detection Method Employed for Data Groups; Table S5. Upper Boundary Mercury Values of Environmental Samples Collected near E-Waste Sites; Table S6. Upper Boundary Mercury Values of Biomarker Samples from Populations Involved with E-Waste.
