# Supplementary material for: Systematic Review and Meta-Analysis of Mercury Exposure among Populations and Environments in Contact with Electronic Waste
Source: Int J Environ Res Public Health. 2022 Sep 20;19(19):11843. doi: 10.3390/ijerph191911843 (PMC9564538; doi:10.3390/ijerph191911843)
Supplement: Supplementary file 1 [file ijerph-19-11843-s001.zip › ijerph-1924459-supplementary.pdf]

Supplementary Information DOC file – Tables and Figures

Systematic Review and Meta-Analysis

Systematic Review and Meta-Analysis of Mercury Exposure Among Populations and Environments in Contact with Electronic Waste

Gwen Aubrac [1], Ashley Bastiansz [2] and Niladri Basu [2,*]

[1] Bieler School of Environment, McGill University, Montreal, QC H3A 2A7, Canada
[2] Faculty of Agricultural and Environmental Sciences, McGill University, Montreal, QC H9X 3V9, Canada
[*] Correspondence: niladri.basu@mcgill.ca

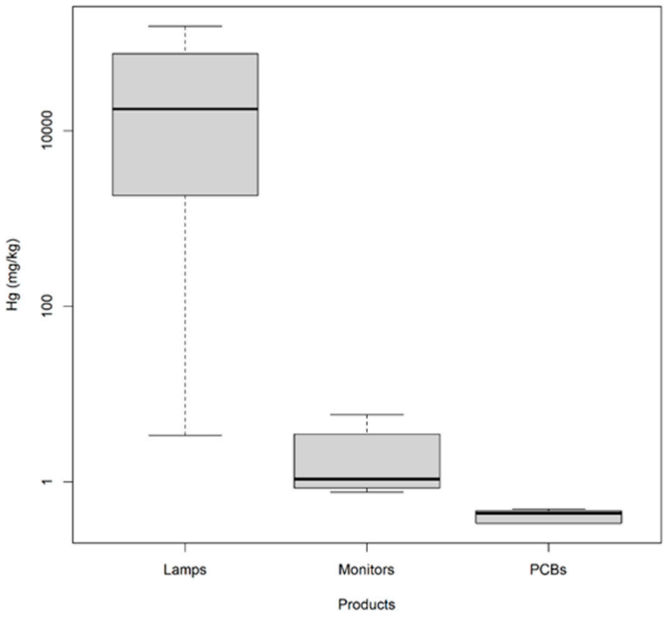

Figure S1. Upper Boundary Values of Mercury in E-Waste Products.

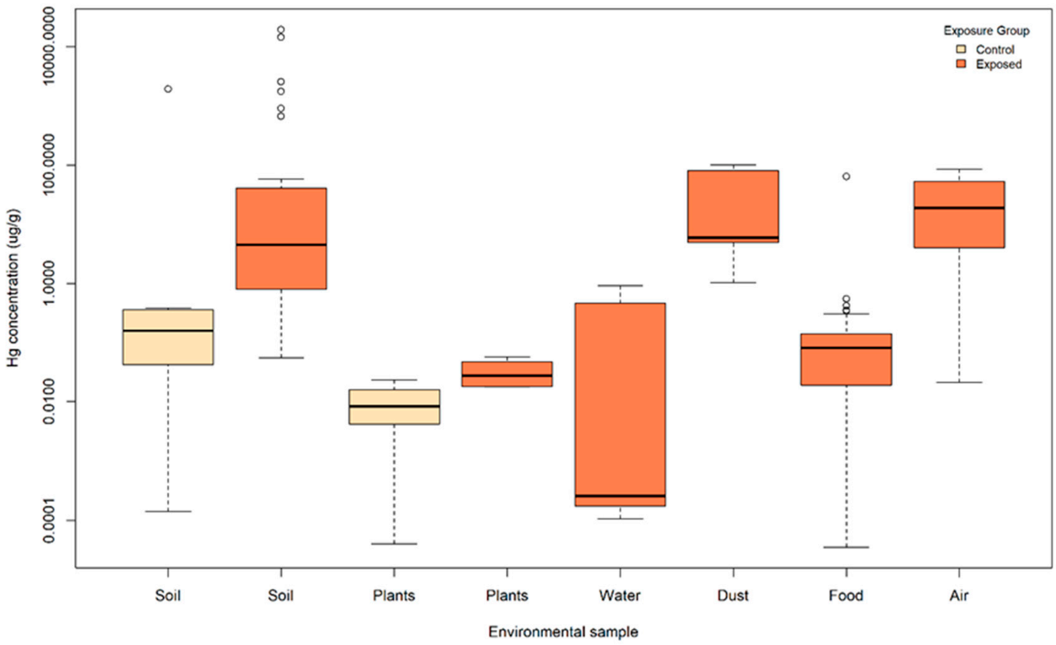

Figure S2. Upper Boundary Values of Mercury in Environmental Samples Exposed to E-Waste.

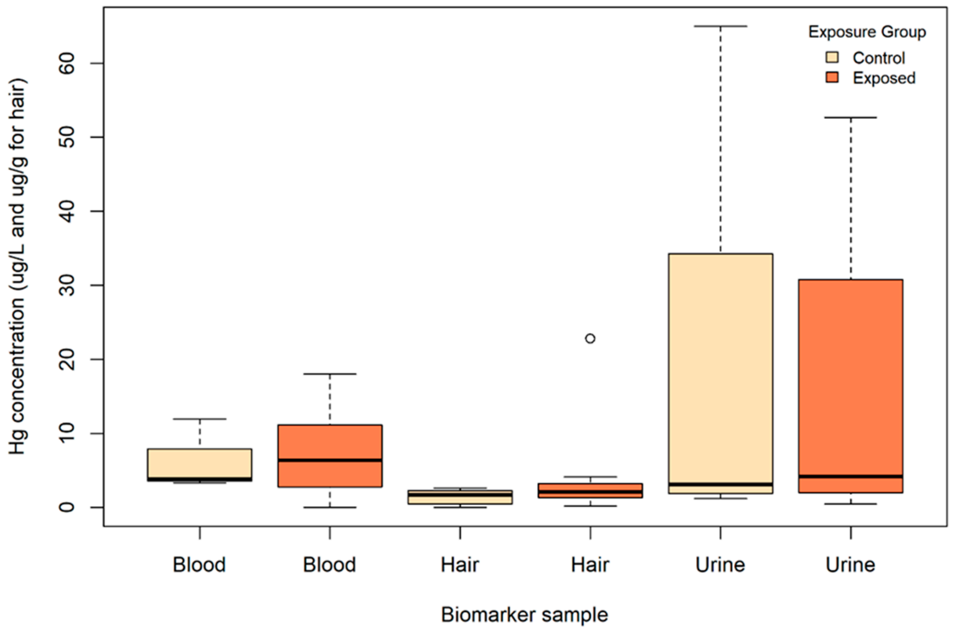

Figure S3. Upper Boundary Values of Mercury in Human Biomarkers of Populations Exposed to E-Waste.

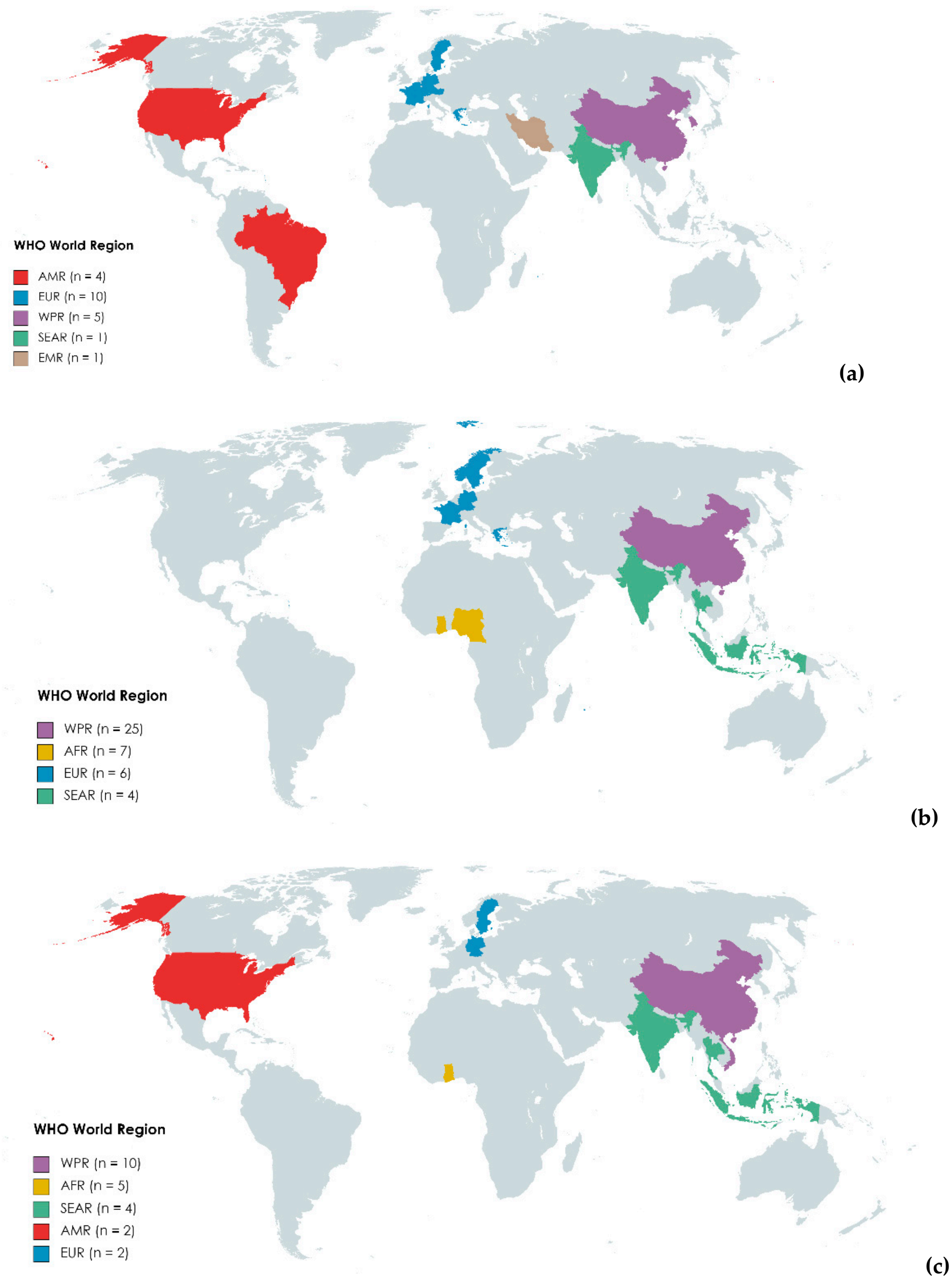

Figure S4. Geographic Location of Studies. (a) Location of Studies on Mercury in E-Waste Products. 2 studies reported no data on sampling country. (b) Location of Studies on Mercury in Environments Contaminated by E-Waste. (c) Location of Studies on Mercury in Biomarkers of Populations Exposed to E-Waste. Maps were created using mapchart.net.

Table S1. Risk of Bias Assessment Methodology.

Item	Description	Assessment
1	Measurement instrument reported	Not mentioned (0)<br>Inadequately mentioned (1)<br>Clearly presented (2)
2	Accuracy through use of reference materials	Not mentioned or not used (0)<br>Inadequately mentioned (1)<br>Clearly presented (2)
3	Precision through use of replicate measures	Not mentioned or no replicates (0)<br>Inadequately mentioned (1)<br>Clearly presented use of replicates (2)
4	LOD reported and acceptable	LOD not mentioned (0)<br>LOD inadequately mentioned (1)<br>LOD clearly presented (2)
5	Selection method (convenient/random)	Sampling method not mentioned (0)<br>Convenient sampling (1)<br>Random sampling (2)
6	Sample size	0-20 or not mentioned (0)<br>20-50 (1)<br>50+ (2)
7	Use of an appropriate control group	No control group (0)<br>Inadequately mentioned or inappropriate control group (1)<br>Adequate and clearly presented control group (2)
8	Demographics reported	Demographics not reported (0)<br>Demographics poorly reported (1)<br>Demographics thoroughly reported (2)
9	Mercury exposure characteristics reported	No mention of exposure characteristics (0)<br>Brief mention of exposure characteristics (1)<br>Thorough mention of exposure characteristics (2)
10	Key descriptive measures reported	No description of study site and methods (0)<br>Poor description of study site and methods (1)<br>Thorough presentation of study site & methods (2)

Table S2. Methodology for Conversion from Wet Weight to Dry Weight.

Study	Sample Media	Water Content (%)	Source
Steinhausen et al. (2020)	Fish (Mugil cephalus, Mugil curema, Trachinotus ovatus, Pseudotolithus senegallus, Scomber colias)	70	Silva et al., 2008
Zhao et al. (2010)	Vegetables (Chinese mustard, green grocery, and green beans)	95	"Hydrate Your Body with High Water Content Fruits and Vegetables," 2016
	Pulses (soybean)	65	Karkle & Beleia, 2010
	Rice	14	*Measuring Moisture Content - IRRI Rice Knowledge Bank*, n.d.
	Hen eggs	74	*Structure of the Egg - Incubation and Embryology - University of Illinois Extension*, n.d.
	Chicken	65	*Water in Meat and Poultry Products*, n.d.
	Pork	75	*Water in Meat and Poultry Products*, n.d.
	Shellfish (mussels)	96	Harbach et al., n.d.

Table S3. List of Included Studies.

key	title	author	year
AMPO2020	Gastric bioaccessibility and human health risks associated with soil metal exposure via ingestion at an E-waste recycling site in Kumasi, Ghana	Amponsah et al.	2020
ANSE2021	Mobility, spatial variation and human health risk assessment of mercury in soil from an informal e-waste recycling site, Lagos, Nigeria	Anselm et al.	2021
CARL2021	Metal Exposures, Noise Exposures, and Audiometry from E-Waste Workers in Agbogbloshie, Ghana	Carlson et al.	2021
CEBA2017	Metal Exposures at three U.S. electronic scrap recycling facilities	Ceballos et al.	2017
CHEN2010	Study on adverse impact of e-waste disassembly on surface sediment in East China by chemical analysis and bioassays	Chen et al.	2010
DART2017	Essential and non-essential trace elements among working populations in Ghana	Dartey et al.	2017
DEFA2020	Is mercury in fluorescent lamps the only risk to human health? A study of environmental mobility of toxic metals and human health risk assessment	De Farias et al.	2020
DECH2018	Urinary Mercury Levels Among Workers in E-waste Shops in Nakhon Si Thammarat Province, Thailand	Decharat et al.	2018
FOSU2017	Heavy metals concentration and distribution in soils and vegetation at Korle Lagoon area in Accra, Ghana	Fosu-Mensah et al.	2017
FU2008	High levels of heavy metals in rice (Oryza sativa L.) from a typical E-waste recycling area in southeast China and its potential risk to human health	Fu et al.	2008
GERD2021	Metal exposure of workers during recycling of electronic waste: a cross-sectional study in sheltered workshops in Germany	Gerding et al.	2021
GUO2009	Heavy Metal Contamination from Electronic Waste Recycling at Guiyu, Southeastern China	Guo et al.	2009
HA2009	Contamination by trace elements at e-waste recycling sites in Bangalore, India	Ha et al.	2009
HAN2019	Heavy metals in soil contaminated through e-waste processing activities in a recycling area: Implications for risk management	Han et al.	2019
HOBO2017	Recycling oriented comparison of mercury distribution in new and spent fluorescent lamps and their potential risk	Hobohm et al.	2017

HOLG2018	Analysis of the metal content of small-size Waste Electric and Electronic Equipment (WEEE) printed circuit-boards part 1: Internet routers, mobile phones and smartphones	Holgersson et al.	2018
JANG2006	Evaluation of metal leaching from end-of-life laptop computers using the TCLP and other standard leaching tests	Jang et al.	2006
JUCH2013	Disassembly and characterization of liquid crystal screens	Juchneski et al.	2013
JULA2014	Formal recycling of e-waste leads to increased exposure to toxic metals: an occupational exposure study from Sweden	Julander et al.	2014
KAYL2022	Metal leaching from Lithium-ion and Nickel-metal hydride batteries and photovoltaic modules in simulated landfill leachates and municipal solid waste materials	Kayla-Kilgo et al.	2022
KELE2018	Efficiency of the air-pollution control system of a lead-acid-battery recycling industry	Kelektsoglou et al.	2018
KIM2013	Analytical Study on the RoHS of Plastic from Waste Electrical and Electronic Appliances	Kim et al.	2013
KOLI2014	Assessment of toxic metals in waste personal computers	Kolias et al.	2014
KUNTA2020	Exposure to Heavy Metals in Electronic Waste Recycling in Thailand	Kuntawee et al.	2020
KYER2018	Contamination and health risk assessment of exposure to heavy metals in soils from informal e-waste recycling site in Ghana	Kyere et al.	2018
KYER2016	Spatial assessment of soil contamination by heavy metals from informal electronic waste recycling in Agbogbloshie, Ghana	Kyere et al.	2016
LAU2014	Human health risk assessment based on trace metals in suspended air particulates, surface dust, and floor dust from e-waste recycling workshops in Hong Kong, China	Lau et al.	2014
LECL2018	Improving the work environment in the fluorescent lamp recycling sector by optimizing mercury elimination	Lecler et al.	2018
LI2011	Heavy metal contamination of surface soil in electronic waste dismantling area: Site investigation and source-apportionment analysis	Li et al.	2011
LI2016	Generation and detection of metal ions and volatile organic compounds (VOCs) emissions from the pretreatment processes for recycling spent lithium-ion batteries	Li et al.	2016
LI2006	TCLP heavy metal leaching of personal computer components	Li et al.	2006
LI2020c	Influence of metals from e-waste dismantling on telomere length and mitochondrial DNA copy number in people living near recycling sites	Li et al.	2020

LI2020d	The sustaining effects of e-waste-related metal exposure on hypothalamus-pituitary-adrenal axis reactivity and oxidative stress	Li et al.	2022
LIAN2015	Human exposure to mercury in a compact fluorescent lamp manufacturing area: By food (rice and fish) consumption and occupational exposure	Liang et al.	2015
LIM2011	Potential environmental impacts of light-emitting diodes (LEDs): metallic resources, toxicity, and hazardous waste classification	Lim et al.	2011
LIN2017	Decreased vaccine antibody titers following exposure to multiple metals and metalloids in e-waste-exposed preschool children	Lin et al.	2017
LIU2016	The Distribution and Health Risk Assessment of Metals in Soils in the Vicinity of Industrial Sites in Dongguan, China	Liu et al.	2016
LIU2013	Heavy metals and organic compounds contamination in soil from an e-waste region in South China	Liu et al.	2013
LONG2021	Heavy Metal Tolerance Genes Associated With Contaminated Sediments From an E-Waste Recycling River in Southern China	Long et al.	2021
LUO2018a	A real scale phytoremediation of multi-metal contaminated e-waste recycling site with Eucalyptus globulus assisted by electrical fields	Luo et al.	2018
LUO2018b	Using solar cell to phytoremediate field-scale metal polluted soil assisted by electric field	Luo et al.	2018
LUO2018c	Heavy metal remediation with Ficus microcarpa through transplantation and its environmental risks through field scale experiment	Luo et al.	2018
LUO2016	Phytoremediation efficiency OF CD by Eucalyptus globulus transplanted from polluted and unpolluted sites	Luo et al.	2016
MARA2013	Qualitative and quantitative determination of heavy metals in waste cellular phones	Maragkos et al.	2013
MEST2005	Characterization of the hazardous components in end-of-life notebook display	Mester et al.	2005
MORF2007	Metals, non-metals and PCB in electrical and electronic waste--actual levels in Switzerland	Morf et al.	2007
NARA2021	Bioremediation of noxious metals from e-waste printed circuit boards by Frankia	Narayanasamy et al.	2021
NEUB2018	Testing Electronic Device Components for RoHS/WEEE Compliance Using Microwave Digestion and ICP-OES	Neubauer	2018
NFOR2021a	Effects of Electronic and Electrical Waste-Contaminated Soils on Growth and Reproduction of Earthworm (Alma nilotica)	Nfor et al.	2021
NI2014	Hair mercury concentrations and associated factors in an electronic waste recycling area, Guiyu, China	Ni et al.	2014

RIOS2021	Methylmercury Measurements in Dried Blood Spots from Electronic Waste Workers Sampled from Agbogbloshie, Ghana	Rios et al.	2021
SALH2011	Assessment of removal of components containing hazardous substances from small WEEE in Austria	Salhofer & Tesar	2011
SAVV2017	Toxicity assessment and feasible recycling process for amorphous silicon and CIS waste photovoltaic panels	Savvilotidou et al.	2017
SAVV2014	Determination of toxic metals in discarded Liquid Crystal Displays (LCDs)	Savvilotidou et al.	2014
SCHE2018	Biomonitoring of Metals, Polybrominated Diphenyl Ethers, Polychlorinated Biphenyls, and Persistent Pesticides in Vietnamese Female Electronic Waste Recyclers	Schecter et al.	2018
SING2020	Toxicity evaluation of E-waste plastics and potential repercussions for human health	Singh et al.	2020
SNOW2021	Characterization of inhalation exposure to gaseous elemental mercury during artisanal gold mining and e-waste recycling through combined stationary and personal passive sampling	Snow et al.	2021
SOET2020	Chronic exposure to heavy metals from informal e-waste recycling plants and children's attention, executive function and academic performance	Soetrisno & Delgado-Saborit	2020
SRIG2016	Multiple elemental exposures amongst workers at the Agbogbloshie electronic waste (e-waste) site in Ghana	Srigboh et al.	2016
STEI2021	Heavy metals in fish nearby electronic waste may threaten consumer's health. Examples from Accra, Ghana	Steinhausen et al.	2021
TAGH2014	Determining heavy metals in spent compact fluorescent lamps (CFLs) and their waste management challenges: some strategies for improving current conditions	Taghipour et al.	2014
TANG2015a	Mercury levels and estimated total daily intakes for children and adults from an electronic waste recycling area in Taizhou, China: Key role of rice and fish consumption	Tang et al.	2015
TANG2010	Heavy metal and persistent organic compound contamination in soil from Wenling: an emerging e-waste recycling city in Taizhou area, China	Tang et al.	2010
TANG2016	Polybrominated diphenyl ethers (PBDEs) and heavy metals in road dusts from a plastic waste recycling area in north China: implications for human health	Tang et al.	2016
TANG2015b	Contamination and risk of heavy metals in soils and sediments from a typical plastic waste recycling area in North China	Tang et al.	2015
TZOR2020	Trace metals' contamination in water and soils in the vicinity of a small–medium waste electrical and electronic equipment recycling plant	Tzoraki et al.	2020

WANG2015b	Distribution of metals and brominated flame retardants (BFRs) in sediments, soils and plants from an informal e-waste dismantling site, South China	Wang et al.	2015
WITT2017	Pilot study on the internal exposure to heavy metals of informal-level electronic waste workers in Agbogbloshie, Accra, Ghana	Wittsiepe et al.	2017
WU2017	Ecological effects of soil properties and metal concentrations on the composition and diversity of microbial communities associated with land use patterns in an electronic waste recycling region	Wu et al.	2017
WU2018b	Regional risk assessment of trace elements in farmland soils associated with improper e-waste recycling activities in Southern China	Wu et al.	2018
WU2021	Field study on the soil bacterial associations to combined contamination with heavy metals and organic contaminants	Wu et al.	2021
XU2018	Effects of soil properties, heavy metals, and PBDEs on microbial community of e-waste contaminated soil	Xu et al.	2018
XUE2021	Cobalt exposure increases the risk of fibrosis of people living near E-waste recycling area	Xue et al.	2021
ZHAN2014	Risk assessment of polychlorinated biphenyls and heavy metals in soils of an abandoned e-waste site in China	Zhang et al.	2014
ZHAN2020b	Exposure to multiple heavy metals associate with aberrant immune homeostasis and inflammatory activation in preschool children	Zhang et al.	2020
ZHAO2010	Concentrations of selected heavy metals in food from four e-waste disassembly localities and daily intake by local residents	Zhao et al.	2010
ZHAO2015	Levels and ecological risk assessment of metals in soils from a typical e-waste recycling region in southeast China	Zhao et al.	2015
ZIMM2014	Occupational exposure in the fluorescent lamp recycling sector in France	Zimmermann et al.	2014

Table S4. Mercury Detection Method Employed for Data Groups.

Data Group	MA	ICP	ICP-OES	ICP-MS	ICP-AES	ICP-ES	EDX-FS	CVAAS	AFS	Not Specified	Other
Percent Employed in Mercury in E-Waste Products Studies	5.5	0.8	5.5	37.8	5.5	0	5.5	15.8	5.5	5.5	5.5
Percent Employed in Mercury in Environments Exposed to E-Waste Studies	5.7	0	2.4	10.9	5.2	6.2	5.7	21.8	28.9	0	13.3
Percent Employed in Mercury in Biomarkers of Populations Exposed to E-Waste Studies	12.5	0	0	28.1	0	0	0	25	1.6	4.7	28.1

Table S5. Upper Boundary Mercury Values of Environmental Samples Collected near E-Waste Sites.

**exposure group**	**upper boundary value**									
**1**	sample media category	unit	total samples	geometric mean	median	mean	IQR	minimum	maximum	SD
	soil & sediment	mg/kg	618	8.70	4.55	1248.67	31.47	0.06	19400.00	4197.22
	water	mg/kg	37	0.00	0.00	0.23	0.23	0.00	0.93	0.46
	dust	mg/kg	65	12.05	5.97	39.09	76.57	1.04	102.00	48.65
	food or animals	mg/kg	113	0.06	0.08	2.71	0.12	0.00	64.80	12.94
	air	μg/m3	768	8.55	19.00	29.20	49.23	0.02	86.70	31.51
	plants	mg/kg	182	0.03	0.03	0.03	0.02	0.02	0.06	0.02
	other	mg/kg	N.A.	N.A.	N.A.	N.A.	N.A.	N.A.	N.A.	N.A.
**2**	soil & sediment	mg/kg	369	0.62	1.50	3.67	2.96	0.00	30.02	6.70
	dust	mg/kg	23	0.66	0.67	0.67	0.14	0.53	0.81	0.20
	food or animals	mg/kg	13	0.27	0.14	0.56	0.67	0.10	1.44	0.77
	air	μg/m3	N.A.	N.A.	N.A.	N.A.	N.A.	N.A.	N.A.	N.A.
	plants	mg/kg	14	N.A.	N.A.	0.00	N.A.	N.A.	N.A.	N.A.

**3**	soil & sediment	mg/kg	15	N.A.	N.A.	0.13	N.A.	N.A.	N.A.	N.A.
	food or animals	mg/kg	10	0.27	0.29	0.29	0.10	0.19	0.39	0.14
	air	µg/m3	6	5.19	5.20	5.20	0.40	4.80	5.60	0.57
**4**	soil & sediment	mg/kg	44	0.19	0.16	242.02	0.31	0.00	1935.00	684.07
	water	mg/kg	N.A.	N.A.	N.A.	N.A.	N.A.	N.A.	N.A.	N.A.
	dust	mg/kg	4	0.05	N.A.	0.08	N.A.	N.A.	N.A.	N.A.
	food or animals	mg/kg	57	0.77	18.21	18.21	18.19	0.02	36.40	25.73
	air	µg/m3	112	0.45	0.53	0.27	0.53	0.26	0.79	0.37
	plants	mg/kg	26	0.00	0.01	0.01	0.01	0.00	0.02	0.01

Table S6. Upper Boundary Mercury Values of Biomarker Samples from Populations Involved with E-Waste.

**exposure group**	**upper boundary value**									
**1**	sample media category	unit	total samples	geometric mean	median	mean	IQR	minimum	maximum	SD
	blood	μg/L	399	2.20	6.40	7.46	8.40	0.00	18.00	6.41
	serum	μg/L	128	1.84	1.85	1.85	0.15	1.70	2.00	0.21
	urine	μg/L	273	0.32	0.80	1.72	2.00	0.00	4.40	1.80
	urine adjusted for creatinine	μg/g creatinine	287	5.74	4.20	18.01	28.76	0.46	52.64	23.00
	hair	μg/g	151	2.03	2.10	4.54	1.43	0.18	22.80	7.47
**2**	blood	μg/L	380	2.23	6.73	10.19	10.09	0.00	29.82	10.93
	serum	μg/L	26	15	N.A.	15.00	N.A.	N.A.	N.A.	N.A.
	urine	μg/L	11	0.001	N.A.	0.00	N.A.	N.A.	N.A.	N.A.
	urine adjusted for creatinine	μg/g creatinine	26	6.8	N.A.	6.8	N.A.	N.A.	N.A.	N.A.
	hair	μg/g	227	0.72	1.48	1.89	1.56	0.01	3.98	1.53
**3**	blood	μg/L	116	9.36	7.73	10.54	6.05	5.90	18.00	6.52
	serum	μg/L	65	2.9	N.A.	2.9	N.A.	N.A.	N.A.	N.A.
	urine	μg/L	10	1.6	N.A.	1.6	N.A.	N.A.	N.A.	N.A.

	urine adjusted for creatinine	µg/g creatinine	132	5.20	3.10	18.10	16.70	1.20	65.00	31.28
	blood	µg/L	288	4.93	3.85	5.75	2.20	3.34	11.94	4.14
**4**	urine	µg/L	60	0.79	0.80	0.80	0.09	0.71	0.89	0.13
	urine adjusted for creatinine	µg/g creatinine	96	3.74	12.37	12.37	11.79	0.58	24.16	16.67
	hair	µg/g	213	2.43	2.44	2.44	0.15	2.29	2.58	0.21
